# Reconceptualizing solidarity as power from below

**DOI:** 10.1007/s11098-022-01845-y

**Published:** 2022-07-20

**Authors:** Robin Zheng

**Affiliations:** Department of Philosophy, University of Glasgow, 69 Oakfield Ave, Glasgow, G12 8LP Scotland, UK

**Keywords:** Solidarity, Power, Social change, Resistance

## Abstract

I propose a new concept of solidarity, which I call “solidarity from below,” that highlights an aspect of solidarity widely recognized in popular uses of the term, but which has hitherto been neglected in the philosophical literature. Solidarity from below is the collective ability of otherwise powerless people to organize themselves for transformative social change. I situate this concept with respect to four distinct but intertwined questions that have motivated extant theorizing about solidarity. I explain what it means to conceptualize solidarity from below as a form of power, rather than as a feeling, disposition, duty, or scheme of social arrangements. Finally, I suggest that the moral-relational aspects of solidarity emerge secondarily from the process of collective power, and not the other way around.

Solidarity is a many-splendored thing. There is no question that solidarity involves some kind of unity, cohesion, or coordination—some form of *togetherness*. But solidarity is variously conceived of as a feeling, sentiment, or disposition; a type of action; a relationship; or a scheme of social arrangements. These are examples of what I will call “moral-relational” concepts of solidarity. Of late there has been a resurgence of philosophical interest in solidarity, particularly in the wake of the COVID-19 pandemic, which has thrown into sharp relief just how truly interconnected our lives and interests are. Solidarity has been widely promoted as a solution to this unprecedented crisis, particularly by global institutions such as the United Nations and World Economic Forum, which took up the motto of “Shared Responsibility, Global Solidarity,” established Solidarity Response Funds, and celebrated displays such as people singing songs together on balconies and applauding healthcare workers (United Nations, 2020; Broome, 2020). Indeed, solidarity has long been studied by philosophers and social scientists alike, in both purely descriptive and normatively prescriptive registers.

In article, however, I take a different tack. First, I offer a novel taxonomy of different uses of the term “solidarity” which remaps the conceptual space by tracing the origins of the distinct philosophical questions for which a notion of solidarity is needed. Second, I spotlight a hitherto neglected dimension of solidarity, which itself remains relatively undertheorized. As Avery Kolers (2016, p. 2) puts it: “[Lack of philosophical interest in theorizing solidarity] is strange, since, in the first place, solidarity is the essential and sometimes the only weapon of all popular politics and justice movements…” While I do not mean to downplay the moral-relational aspects of solidarity, I take up Kolers’ suggestive comment by developing a novel concept of solidarity understood as a form of *collective power*, which I call “solidarity from below.”

Solidarity from below is the collective ability of otherwise powerless people to organize themselves for transformative social change. I begin in Sect. 1 by situating this within the existing philosophical literature on solidarity. Next, in Sect. 2 I conceptualize what it means to say that solidarity is a form of power, and provide some clarification and further elaboration in Sect. 3. I conclude in Sect. 4 by proposing that many other kinds of moral-relational solidarity actually depend on solidarity from below—and not the other way around, as commonly assumed—such that its ethical and political dimensions are in dialectical relationship.

## Theorizing solidarity: four questions

I will not attempt here to provide a full literature review, exhaustive list of existing conceptions of solidarity, taxonomies of its varieties, or detailed history of the idea (see, e.g., Bayertz, 1999; Scholz, 2008; Stjernø, 2009). Instead, I offer a conceptual genealogy that disentangles four distinct but intertwined questions which have motivated theoretical and practical discussions of solidarity:

1. What holds society together?
2. How should we relate to others (in general)?
3. How should we relate to (specific) others (in our own group)?
4. How can we collectively bring about social change?

This will situate my concept of “solidarity from below” as part of an answer to the fourth question.

### What holds society together?

One prominent use of the term “solidarity” as a *sociological* concept derives from 19th-century European social theorists concerned with the disintegration of ‘traditional’ society in the transition to ‘modernity,’ that is, the processes of widespread industrialization, urbanization, and secularization that accompanied the rise of capitalism (Stjernø, 2009). In preindustrial feudal society, peasants regularly relied on one another for reciprocal aid, both in and beyond the family. But these kinship and communal ties were ruptured in the social upheavals that, both materially and ideologically, occasioned a shift away from tradition and community towards ‘rationality’ (in the sense of instrumental means-end thinking, characteristic of modern economics) and individualism. For Auguste Comte, who first coined the term “sociology,” solidarity is the characteristic of human society that makes it an internally organized unity, integrated across a division of labor, which is what enables it to manifest “continuity” over time (Wilson, 1927)—that is, the uniquely human transmission of knowledge, resources, and culture across generations (Stjernø, 2009, pp. 31–32). Members of a society are thus bound together both diachronically and synchronically: they are dependent on (and indebted to) their ancestors, and on each other, for their material survival (Peters, 2014; Stjernø, 2009, p. 32).

Emile Durkheim, the founding father of modern sociology, further develops this idea of solidarity as the ‘glue’ that bonds together members of a society, distinguishing between two types that exemplified the transition to modernity. While “mechanical” solidarity arises from the *homogeneity* of shared cultural norms, beliefs, rituals, and material conditions that characterized members of traditional society, “organic” solidarity arises from the *heterogeneity* of a society in which an increasingly specialized division of labor creates individuals who do *not* share the same material conditions or culture, but who—by the same token—are rendered totally interdependent on one another for survival (Scholz, 2008; Stjernø, 2009). Both Comte and Durkheim were writing in opposition to laissez-faire economic thinking and the Hobbesian theory that society could be held together by a rationally self-interested social contract. Instead, they believed, social order and harmony requires that feelings of solidarity between members of society be actively cultivated, particularly where the sameness of mechanical solidarity has been disrupted (Peters, 2014; Stjernø, 2009). Thus, although the sociological interest in solidarity (carried on by Hechter (1988) and others) is a largely *descriptive* project of explaining social cohesion and order, it has always taken on clearly normative overtones.

### How should we relate to others (in general)?

The normative dimensions of solidarity are highlighted in religious and philosophical traditions that use it as an *ethical* and *political* concept to characterize the relationships, attitudes, and actions we ought to undertake with respect to others. An early precursor to the modern concept of solidarity was that of “brotherhood” or “fraternity,” which emerged in Greco-Roman and medieval Christian communities to denote the bonds that unite religious believers (Peters, 2014; Stjernø, 2009). Over time, solidarity became understood as a compassionate response to the poor and suffering, which calls for collective action beyond what is required by the individual virtue of charity. In particular, 20th-century Roman Catholicism has laid strong emphasis on solidarity—in part due to the tradition of Latin American liberation theology, which articulated the idea of the “preferential option for the poor” in order to highlight structural causes of poverty and assert the poor’s right to self-determination in their struggles against right-wing military repression and U.S. imperialism (Peters, 2014). Solidarity was elevated by Pope John Paul II into a key concept of Catholic teaching, alongside other values like “justice” and the “common good.” Here, solidarity represents the correct “moral and social attitude” between rich and poor: it is “not a diffuse feeling of compassion, but a firm and lasting commitment to the best for all [in which those] who have resources and influence should feel responsibility for the weak and share their resources with them” (Stjernø, 2009, p. 71).

Catholic solidarity is grounded in the universal humanity found equally within all of God’s children. However, secular philosophers have also defended conceptions of universalist human solidarity as expressed in feelings of caring for and standing with others across national borders (Gould, 2004, 2007), of the mutual expectations and responsibility-taking across social difference (Dean, 1996), a relationship of empathetic understanding to be extended as far as practical limits allow, even to non-human animals (Harvey, 2007), and a radical openness to acknowledging indefinitely many diverse perspectives (Medina, 2013).

Some of these ethical insights—particularly around justice and the common good—are also captured in political theory, where solidarity is appealed to as the normative basis for the (European) welfare state.^1^ The early Roman legal concept *in solidum* referred to jointly held debt, liability, or obligation (Brunkhorst, 2005). Likewise, one of the earliest political uses of the term “solidarity” appeared in Charles Fourier’s *Theorie de l’Unité (*a tract of utopian socialism), where it refers not only to a general feeling of community and disposition to help those in need, but also more specifically to a scheme of social insurance and collective repayment, a guaranteed minimum income, and public support for (male) family providers (Stjernø, 2009, p. 28). Later, Marxist social democrats such as Karl Kautsky and Eduard Bernstein explicitly articulated the idea that social arrangements should conform to the principle of solidarity, in which individual freedom is balanced against the common good (Stjernø, 2009). Most famously, Jürgen Habermas (1990, p. 47) has argued that respecting universal rights and procedural adherence to justice entails simultaneously promoting particular communities and feelings of solidarity: “[Solidarity] is rooted in the realization that each person must take responsibility for the other because as associates all must have an interest in the integrity of their shared life context in the same way. Justice conceived deontologically requires solidarity as its reverse side.” Solidarity motivates people to contribute their fair share to social arrangements which maintain the public goods needed to sustain the welfare of the entire community.

In declaring that the relationship between justice and solidarity is “not so much of two moments that supplement each other as of two aspects of the same thing” (47), Habermas’s work exemplifies the use of solidarity as the bridge concept mediating between inviolable individual rights and wider community obligations (Scholz, 2008), between the Kantian conception of the autonomous, self-determining individual and the Hegelian insight that this is only made possible through collective socialization (Carrabregu, 2016).^2^ Solidarity is also importantly (if not always recognized to be) connected to ideals of *care,* which take center place in feminist care ethics and political theory. Thus theorists like Iris Marion Young (2002) and Ann Ferguson (2009) have also proposed solidarity-based paradigms of justice which recognize socially differentiated publics and a universal caregiver citizen. Other theorists have used the concept of solidarity (sometimes called “political solidarity”) to theorize the necessary relations of mutual respect, trust, loyalty, obligation and support that connect citizens of a polity, applying them to diverse issues such as multiracialism and multiculturalism (Hooker, 2009), public health (Krishnamurthy, 2013), and climate change (Doan & Sherwin, 2016).

### How should we relate to (specific) others (in our own group)?

A different approach to solidarity traces its origins to Max Weber’s influential discussion of “social closure,” that is, the processes by which groups draw boundaries delineating between “we” and “them,” thereby reserving goods (e.g. honor, material resources) for some while excluding others (Stjernø, 2009, p. 28). This kind of *group* solidarity—in both its descriptive and normative dimensions—has been well-theorized by contemporary philosophers. Tommie Shelby’s (2005) celebrated work on Black solidarity outlines four features of robust group solidarity: feelings of subjective identification with other group members, special concern for and actions toward their welfare, shared values and goals, loyalty, and mutual trust.^3^ To this may be added Joel Feinberg’s (1968) conception of solidarity as involving vicarious pride and shame, integrated interests, and the experience of sharing a “common lot” (or “linked fate,” from the literature on racial identity). A substantial body of work also addresses issues of racial, gender, and other social identities, which is typically considered a key basis for group solidarity (Alcoff, 2005; Blum, 2007; McGary, 2012). Alison Weir (2013) has also argued that some kind of solidaristic perception is a necessary part of the process by which women are able to construct their interests and identities as women at all.

The phenomenon of group solidarity has been sharply criticized, however. Audre Lorde (1984/2007), bell hooks (1984), Chandra Mohanty (2003) and others have called out the presumed solidarity of “sisterhood” amongst feminists for obscuring the oppressive racial, class, and heterosexual hierarchies that subordinate some women to others (see also Sect. 2.2). A large body of feminist and critical race theory has struggled with the dilemma of trying to establish some sort of solidarity that could serve as the basis for collective action, without grounding it in interests and experiences that exclude more marginalized members of the group, or a set of essential criteria delineating who counts as a real ‘woman’ or ‘Black’ or ‘Latina’ (etc.) (Spelman, 1988; Stone, 2004; Weir, 2013). While Anthony Appiah (1989) contends that racial solidarity necessarily entails this kind of objectionable essentialist assumption of inherent or cultural difference, Shelby (2005) and others have argued compellingly that it does not (see also Gooding-Williams, 2009; Howze & Weberman, 2001; McPherson & Shelby, 2004; Rogers, 2002). Critics of identity politics have also argued that group solidarity is detrimental to progressive aims. However, theorists such as Young (1990), Dean (1996), and Linda Martín Alcoff (2005) have countered with more sophisticated conceptions of democracy and group identity that rebut these charges (see also Doan & Sherwin, 2016; Stanley, 2014).

On the other hand, there is no doubt that appeals to group solidarity *can* be deeply pernicious, as in Clarence Thomas’s notorious declaration that his Supreme Court nomination trial was a ‘high-tech lynching’, which rallied the loyalty and special concern of Black racial solidarity in his defense—at the expense of Anita Hill (a Black woman), who was vilified for testifying that Thomas (a Black man) had sexually harassed her repeatedly (Collins, 1990/2000; Dean, 1996; Morrison, 1992).^4^

We should not be surprised that forms of group solidarity based on race, class, and other such social identities have been so controversial in a world that is so profoundly stratified by them. Unjust social hierarchies generate cross-cutting conflicts of interest along all these dimensions, at least in the short-term, that divide people from one another. Instead of trying to adjudicate these debates here, then, let me instead pivot to the final question. Clearly, the reason why identity-based solidarities—as opposed to group solidarity amongst families, sports team fans, and so on—have been the subject of such intense discussion is due to the ways that people are *oppressed* on the basis of their race, gender, and so on. Group solidarity is particularly important for these constituencies because it is a tool for resisting and overcoming oppression.

### How can we collectively bring about social change?

This brings us to the idea of solidarity that has long been in use within the *socialist* tradition, though of course this usage has spread far beyond the socialist movement. What I will call “solidarity from below” is a concept that falls within this lineage, though it is informed by much a much wider set of influences.

Historically, the early Christian idea of fraternity shifted in meaning from purely religious to more political connotations during the French Revolution, where it took its place in the famous call for *liberté, égalité, fraternité!* In this context, fraternity was understood as a tool of the revolution. Stjernø (2009) writes:

Feelings of brotherhood were to be a means of realising equality, and the Jacobins established *societies of brotherhood* among revolutionaries to achieve the goals of the revolution…Occupational differences and differences in the financial status of revolutionaries were downplayed, and the concept was part of the practical programme implemented to change society and its institutions. (27)

By subverting class hierarchies, however temporarily, French revolutionaries were able to momentarily link up the otherwise divergent interests of the emerging middle class (the *bourgeoisie*) and the poorer classes, consolidating a powerful force that successfully overcame the centuries-old rule of the French monarchy.

The idea of “brotherhood” and “unity” amongst the oppressed and exploited soon evolved into the idea of working-class solidarity, which has played a starring role in the revolutionary Marxist tradition.^5^ Although Marx and Engels rarely used the term “solidarity,” they regularly invoked the importance of worker unity, alongside rhetorical locutions such as “fraternal feelings” and “being brothers” (Stjernø, 2009, p. 46). Just like the social theorists discussed earlier, Marx *qua* social scientist took up the idea of solidarity in both a descriptive vein, as part of his broader theory of “scientific socialism,” as well as a normative one, as part of his political program. Marx observed that even while the rise of capitalism destroyed traditional social bonds, it also generated new ties between the increasing numbers of workers who were thrown into close proximity with one another on the factory floor. For Marx, capitalism had planted the seeds of its own destruction, by producing a new agent of social change: the working class (or *proletariat*). For the first time in history, the exploited classes had the capacity—through their concentration in factories, where they could recognize their shared material interests and organize themselves—to achieve a collective power strong enough to overthrow their exploiters. Subsequent theorists and practitioners would call this the power of “solidarity.”^6^

Stjernø argues that, for Marx, these normatively-laden affective feelings of unity between workers are cast in a primarily instrumental light. Moreover, for Marx and Engels, unity is to be generated *through the struggle itself,* that is, through a distinctive political practice (Stjernø, 2009, pp. 44–47)—an idea I sketch further in Sect. 4. Subsequent philosophers have also taken up this idea. Indeed, this is precisely the thrust behind pragmatic theories of racial solidarity advanced by Eddie Glaude (2000), Tommie Shelby (2005), and Robert Gooding-Williams (2009); Mabogo More (2009) has also argued that collective racial solidarity is the only feasible way to rectify collectively-experienced racial injustice. In the feminist tradition, Chandra Mohanty (2003, p. 7) defines solidarity as a *practice* of “praxis-oriented, active political struggle” that forges “communities of people who have chosen to work and fight together.” These conceptions of solidarity are also often expressed in terms of feminist coalition-building*,* for example in what Bernice Johnson Reagon (1981/2000) and Nira Yuval-Davis (1999) refer to, respectively, as “coalition politics” or “transversal politics” (see also Bain, 2017; Collins, 2017).

Sally Scholz (2008) has offered a theory of this kind of “political solidarity”^7^ amongst participants in social movements for change. For Scholz, groups committed to engaging each other in political solidarity thereby incur specific sets of moral obligations with respect to their fellow members of the solidary group, the shared goal of the movement, and those who are not part of the movement. Avery Kolers (2016) has also developed a detailed (non-instrumental)^8^ account of the moral duty of solidarity that requires more privileged groups to defer to the interests and judgment of oppressed groups. Geographer David Featherstone (2012) has also developed an influential account of solidarity as a “relation forged through political struggle which seeks to challenge forms of oppression,” emphasizing that it is a practice that can emerge  through ‘pressure from without’ and ‘from below’. My proposed concept of “solidarity as below” falls in line with these instrumental, pragmatic, and teleological ways of thinking about solidarity. However, existing accounts do not explicit theorize solidarity as a form of power, as I will do shortly, following Allen (1999).

Conceiving of solidarity as a form of collective power captures something that is importantly missing from the existing concepts of solidarity that have hitherto populated the literature. Specifically, it elucidates how it is that solidarity is able to bring about radically transformative social change, even when the people coming together in solidarity are often the most vulnerable and deprived. Indeed, solidarity is a powerful force that even the ‘Have Nots’—those without money, guns, or political influence—have got. This is clear from the concluding lyrics of the popular labor anthem “Solidarity Forever.” While an earlier stanza plaintively decries the miserable social conditions borne by workers:

It is we who plowed the prairies, built the cities where they trade

Dug the mines and built the workshops, endless miles of railroad laid

Now we stand outcast and starving 'midst the wonders we have made

But the union makes us strong

the final two plainly extol the real social power generated by the union:

They have taken untold millions that they never toiled to earn

But without our brain and muscle not a single wheel can turn

We can break their haughty power gain our freedom when we learn

That the Union makes us strong

In our hands is placed a power greater than their hoarded gold,

Greater than the might of armies, multiplied a thousand-fold.

We can bring to birth a new world from the ashes of the old

For the union makes us strong.

Moreover, this power is explicitly understood as being directed toward transformative social change: toward birthing “a new world.” In leftist iconography, the ubiquitous ‘solidarity fist’—passed down from early 20th-century Industrial Workers of the World (IWW), anti-Fascists in the 1930s Spanish Civil War, 1960s Black Power activists, and even co-opted by right-wing reactionaries (Kelly, 2012)—is also an unmistakable symbol of power. Extant theories of solidarity, then, ought to be supplemented with a clear understanding of solidarity from below.

## Solidarity as power

The concept of “power” is one of the most highly theorized—and therefore slipperiest—ideas in social and political philosophy, social and critical theory, and the social sciences. It has also received significant attention from activist scholars in social movement and development studies. Power can operate *despotically*, in a manifest contest between conflicting forces, where the powerful overtly coerce or repress the powerless. Or, it can operate *hegemonically*, in which case the powerless willingly behave in ways that serve the powerful, because they have ‘bought into’ the system (Gramsci, 1971; Lukes, 1974/2004; Wright, 2010). Relatedly, power is often conceived of as a thing possessed and ‘wielded’ by a minority of individuals or group, which may be ‘taken’ or ‘wrested’ away by others who lack it. But power is also understood to be something like circulates through a much more diffuse network of various mechanisms via which *all* individuals are subject to, exercise, and are deeply affected—indeed, even produced^9^—by it (Collins, 1990/2000; Foucault, 1990). Power can involve domination and control, or it can be creative and life-affirming (Freire, 1970/2014; hooks, 1984).

### Power-With

Evidently, the concept of power picks out a number of very different things. Here, I adopt Amy Allen’s (1999) version of a taxonomy that distinguishes between *power-over*, *power-to*, and *power-with.*^10^ Allen develops this taxonomy to illuminate three distinct theoretical tasks for which feminists and other critical theorists need a concept of power. It must be noted, however, that these are three interrelated facets of power (which presuppose one another) rather than distinct types or kinds.

The first task is understanding how it is that certain social groups exercise domination over others. *Power-over* is the kind of influence or control wielded by those who have it over those who lack it, which enables the former to (intentionally or unintentionally) constrain the choices of the latter such that they act in ways they otherwise would not (cf. Dahl, 1957). While power-over is not always a bad thing, domination and oppression are particular species of power-over that unjustly harm those who lack it.^11^ Secondly, critical theorists need to make sense of how it is that oppressed people, in spite of being dominated, nevertheless still manage to resist that domination—that is, we need to identify the kind of power developed in processes of *empowerment*.^12^ This is *power-to*: the creative ability of agents to consciously act so as to bring about desired outcomes in the world, in the face of constraints (cf. Holloway, 2002; hooks, 1984). Resistance, specifically, is the exercise of power-to against systems of domination.

Finally, *power-with* is the collective ability of individuals and groups, acting in concert, to attain their ends. For Allen (1999, p. 104), this concept is needed to deal with a dilemma with respect to a problem of (group) solidarity: on the one hand, feminist solidarity grounded in shared experience or common oppression inevitably excludes some women, particularly the most vulnerable; on the other hand, setting aside the category of ‘women’ altogether prevents the recognition of a common cause that can form the basis of collective action. What matters for my purposes is that solidarity from below, as I am conceiving of it, is clearly a form of power-with.

As such, it has several notable features. Allen derives the concept of power-with from Hannah Arendt’s conception of power, which is contrasted with both “violence” and “authority,” as follows:

It is a collective, relational phenomenon that relies on numbers, not implements; it is an end in itself that is, thus, by its very nature legitimate; and, most important, its essence is not command or rule but collaboration and collective action…In Arendt’s view, power emerges from relationships between individuals who are working together for a common goal. (100)

Notably, Allen disagrees with Arendt that power-with is always legitimate, because a group can generate power-with that stems from relations of “receptivity and reciprocity” *within* the group, while using that power-with to dominate and oppress others.^13^ Hence, Allen reserves the concept of “solidarity” to designate the specific form of power-with that is aimed at challenging, subverting, and overturning systems of domination (127).

Allen is concerned specifically with feminist solidarity, which aims at overturning patriarchal domination; her framework deliberately leaves ample room for corresponding forms of solidarity against white supremacy, capitalist exploitation, ableist oppression, and so on. However, I am concerned with *structural injustice* understood as the sum total of distinct but interlocking forms of domination, oppression, and exploitation we face today—in other words, with injustice conceived of as a systemic whole^14^ (Zheng, 2018). Moreover, Allen’s suggestive but cursory remarks on solidarity do not provide a full account of how it is that solidarity can actually bring about its promise of social change (because her theoretical focus lies elsewhere). Hence, I develop the concept of *solidarity from below*—the collective ability of otherwise powerless people to organize themselves for radical, wholesale social transformation—specifically as theory of social change (cf. Zheng, 2022). As a particular kind of solidarity understood in Allen’s terms, solidarity from below is thus a particular type of normatively legitimate power-with.^15^ Like other forms of power-with, the primary source of this solidary power derives from the *numbers* of people engaged in collaboration and collective action, and not their control over coercive instruments (which are the source of power-over). As well-known labor organizer Jane McAlevey succinctly puts it: “The employer class has many sources of power (money, media, and militias, to name a few), but the working class has one: our large *numbers* [emphasis mine]” (McAlevey, 2019a). To this I must add that a second source of solidary power *from below*, which derives in part from numbers but also the very fact of the subordination, exploitation, or oppression to which the powerless masses are subject, is the *necessity* of their labor and acquiescence. Let me now turn to each of these.

### Of numbers and necessity

The powerless, taken as a whole across all forms of systemic oppression, have always had the advantage of *numbers* over the powerful. This is the force behind the rhetoric of ‘the 99% vs. the 1%’. There are several reasons for this. First, as Gene Sharp (1973) emphasizes: in a basic sense, wielding significant power(-over) necessarily relies on the subjection of many. As Hobbes famously pointed out, human beings are fundamentally so equal in their abilities that they each is capable of (in one way or another) inflicting potentially lethal damage on one another. For a single person—the sovereign, say—to have power over others, then, is really for the sovereign to have *under their control* the combined powers of all their troops, ministers, and so on.^16^ Without these, the person who occupies the role of sovereign has only as much power as any other ordinary person. Hence, to be powerful one must in the first instance strip it away from large numbers who are rendered subordinate and, to that extent, powerless. Second, those who possess power tend to hoard it amongst those who resemble themselves, whilst keeping it away from the numerous Other, i.e. the rest of the population who differ at all by race, gender, class, religion, sexuality, nationality, disability, language, and so on. (This is compatible, of course, with sharing a small amount with a limited number of the Other who have been enlisted to serve the ends of the powerful, and hence develop feelings of loyalty or dependence.)

Note that we can hold that the powerless outnumber the powerful even while recognizing particular forms of oppression like ableism, in which the statistically normative able-bodied population outnumbers the minority of people who are disabled. Imagine if we lined up the human population along a multi-dimensional cone with each axis (race, gender, class, etc.) forming one side, such that a small group of people at the tip of the cone possess power along every single axis: white, male, Christian, heterosexual, cisgender, able-bodied, and so on. Every form of difference along any axis locates a person further away from the top; and having power along some axis is still parasitic on the way that power is concentrated overall by a relatively homogenous group of elites in the center. Thus there are many people who are white, male, or otherwise fall on the dominant side of *some* oppression, but only a small number are located at the privileged end of *every* single axis of oppression.^17^

Appreciating the way that power(-over) requires the subjection of many also brings to light the sense in which the powerless play an ineluctably *necessary* role in maintaining existing systems of domination. As Sharp (1973, p. 17) writes: “The power relationship exists only when completed by the subordinates’ obedience to the ruler’s commands and compliance with his wishes.” If subordinates do not comply, then superiors do not successfully exercise power. In a famous passage of his autobiography, Frederick Douglass describes a physical altercation with his former slaveowner Mr. Covey, during which a fellow slave refused Covey’s orders to help him subdue Douglass. Afterward, Covey never “laid the weight of his finger upon [Douglass] in anger” again—vividly demonstrating how his slaves’ compliance was a necessary component of his power over them (Douglass, 1845/1851, p. 67).

In addition to the necessity of their obedience, workers also derive tremendous power from the necessity of their labor. A case from early 2015 demonstrates how this is in principle separable from the power of numbers: a 35-day U.S. government shutdown—the longest in history—was ended just hours after a mere *ten* air traffic controllers called in sick following their second missed paycheck. This is something of a unique example, as air traffic controllers perform highly specialized jobs that require substantial training, and the current pool of workers is at a 30-year low (Jones, 2019; Kaufman & Marsh, 2015). More commonly, especially in a system that values profit-generating over life-sustaining labor, most of the tasks necessary for societal functioning—particularly manual labor and carework—fall to the masses of ordinary workers. As the COVID-19 pandemic has made eminently clear, the frontline essential workers who have continued to work during the crisis (healthcare workers, janitors, grocery store and food service workers, farmworkers, nursing home and childcare workers, garbage truck drivers, and so on) are also some of the least-paid and least-respected. Since this labor is essential but devalued or distasteful to powerful elites, the powerless who are forced perform it thereby gain leverage insofar as they are willing to withhold it.

However, labor is not the only thing that can be withheld in order to gain leverage. By withholding their money via boycotts, consumers gain leverage over specific businesses (which, in turn, can put pressure on local governments). Or, voting blocs can be organized in which people withhold their votes from certain parties or politicians (who, in turn, can exert pressure on particular businesses). Because economic elites require money, political elites require votes, and society as a whole requires essential forms of labor, the powerless can gain leverage if they are properly organized to withhold these. The fact the solidarity can be constituted by these distinctive forms of leverage—which derive essentially from relations of domination and exploitation—is what distinguishes solidarity from below from other forms of collective power.

### The weapons of solidarity

The twin powers of numbers and necessity, however, can be wielded in different ways, via different types of collective action. There is a considerable body of activist and scholarly literature demonstrating that, in order to build the kind of power that can actually generate successful outcomes, groups and movements cannot rely simply on *advocacy* or *mobilizing*, but must focus on *organizing* (e.g., Han, 2014; McAlevey, 2016). Here, I will set aside advocacy—in which people who are ‘experts’ use expensive or elites-only  tactics such as litigation, back-room deals, performing research, making public comments, etc. to bring about policy changes by negotiating with those in power—because it does not essentially depend on the power of solidarity from below. As McAlevey (2016, p. 9) writes: “Advocacy doesn’t involve ordinary people in any real way; lawyers, pollsters, researchers, and communications firms are engaged to wage the battle [and it] fails to use the only concrete advantage ordinary people have over elites: large numbers.”

Mobilizing—that is, the process of ‘activating’ and coordinating large numbers of people for collective actions such as rallies, protests, and petitions—does rely on the power of numbers. At its most powerful, mobilizing can trigger mass protests: intensive, decentralized, and often spontaneous gatherings of people that arise and persist beyond the control of any single individual. For instance, the famous 1960 Greensboro lunch counter sit-ins against racial segregation in the U.S. South spread like wildfire to the rest of the country in a matter of months, prompting the racial integration of dining facilities that very summer. More recently, phenomena such as the #BlackLivesMatter and #MeToo movements clearly demonstrate characteristics of mass protest, in which the sheer number of people participating—which far exceeded the expectations of individuals who initially (and independently) conceived of the phrase “Black Lives Matter” or “Me Too”—broadcast widespread feelings of anger, frustration, and defiance against the status quo.

This kind of “mass defiance,” as sociologists Frances Piven and Richard Cloward (1979) call it, in which people begin to “blame their rulers,” can lead to positively rebellious behavior—blocking the streets, rioting, and so on. With large enough numbers, such disruptive behavior threatens to undermine powerful elites’ control over the system. At the very least, mass protest renders it conceivable that powerful elites *could* or *should* lose control. To maintain order, then, elites can be forced via mass protest to make concessions, such as pulling back an unpopular policy, or terminating prominent individuals from their posts. Mass protests can also drastically shift public discourse in a very short amount of time, as both #BlackLivesMatter and #MeToo have demonstrated (see, e.g., Sawyer & Gampa, 2018).

However, due to their intense and emotional nature, large mobilizations are short-lived and exceptional rather than routine. After the initial defiance, the number of participants declines until the next spark of outrage, as shown by the pattern of #BlackLivesMatter protests that flared up in 2012, 2014, and 2020 (after the high-profile deaths of Trayvon Martin, Mike Brown, Sandra Bland, Breonna Taylor, and George Floyd, among others).

By contrast, *organizing* is the process of building up long-term (often membership-based) associations that unify around concrete demands and a collective decision-making procedure. A labor union, for instance, systematically formulates a list of demands based on the particular working conditions faced by its members, votes formally whether or not to go on strike, and disciplines its members to go out door-knocking, collect signatures, guard picket lines, etc. This structure enables the union to most effectively wield the collective leverage of its members to target and extract highly specific concessions from employer, through periodic bargaining.

Organizing is distinct from mobilizing in several ways. Most importantly, organizing focuses on activating people who are not antecedently committed to collective action, while (routine) mobilizing relies on the regular participation of the ‘usual suspects’. As Han (2014, p. 11) writes, mobilizers work to “get as many people involved as possible, but they do not try to transform or cultivate volunteers’ capacities for further activism;” they provide a menu of typically low-effort actions that interested parties self-select. By contrast, organizers commit to taking responsibility for specific outcomes related to a larger collective goal (e.g., knocking on a target number of doors as part of an electoral campaign), through an escalating series of risky or time- and effort-intensive action (McAlevey, 2020). More importantly, they commit to recruiting developing *new* organizers who will expand the base of ordinary people involved (e.g., forming a team of door-knocking volunteers and training them to develop even more volunteers) (Han, 2014).

At its best, organizing occurs within a finite constituency—a workplace, an apartment building, a church—in which a high enough percentage of people, regardless of their ideological and demographic differences, participates in collective action that forces a powerful decision-maker to act as they want. Workplace stoppages, rent strikes, nonviolent direct action, and electoral majorities all represent organizing victories (McAlevey, 2016, p. 9).

According to McAlevey (2016), Han (2014), and others, what makes organizing arguably much more effective at achieving social change than advocacy and mobilizing is that it is fundamentally based on *empowering* people—that is, in what I would call the process of building up solidarity from below. By involving people in highly social, long-term, sustainable action which progressively cultivates key skills, organizers enable people to take higher-risk, higher-reward collective actions that have a greater chance at delivering concrete outcomes. Mobilizing, by contrast, often leads to burnout on the part of small numbers of dedicated individuals, and fails to leverage the power of numbers and necessity needed to bring about actual social change. Moreover, as Han (2014) demonstrates, organizing is a *transformational* experience for participants, whose effects extend far beyond the duration of a particular campaign (cf. Featherstone, 2012). It teaches people key skills like communication and democratic decision-making, strengthens their political motivations and commitments, and above all, provides them with a first-hand taste of collective power.

Consider the following testimonials from teachers who participated in the 2018 #RedforEd teachers’ strikes that swept through ‘red’ states (i.e., states dominated by the Republican party) such as West Virginia, Oklahoma, and Arizona in deeply conservative U.S. states, which scored previously unthinkable victories. One Arizona teacher reports:

Since the strike, there’s a definite sense of solidarity that wasn’t there before…You used to go in to school, do your thing, and go home. Now if there’s a struggle, we go do something about it because we’re in it together. It’s not just that there are a lot more personal friendships—we saw that we had power (qtd. in Blanc, 2019).

This kind of transformational experience can occur through mobilization as well. In early 2000s Singapore, for instance—a country in which there is no freedom of assembly, and political speech is highly regulated—a small (but prominent) women’s advocacy group found itself in the position of needing to defend itself against a hostile takeover by anti-LGBTQ Christian evangelicals. After weeks of mobilizing which included contacting their networks, speaking to the press, and practicing how to give oral comments, the original members called for an extraordinary general meeting, which attracted three thousand participants. After hours of debate, voting, and waiting, the original core regained control of the leadership. Over a decade later, they recounted the ineffability of this transformational experience. “I mean, how do you describe a feeling? You know, it was happiness, joy, and then—but it’s also this sense of being one of many,” said one. Another stated: “Reliving it now, I’m quite overwhelmed [because] I never wanted anything more in my life… that I’ve worked as hard for, that I really really wanted.” In the words of one particularly memorable account:

I can’t describe the feeling. It was- it- we’re Singaporean; we’ve never experienced democracy in this way before. It was in front of our eyes; we were there. We screamed, we shouted, we voted, we- we protested, we agreed, we cheered, we booed, and then we voted, and then the result was in our favor! It was like, [inhales sharply] this is what democracy feels like!

Thus, it is worth emphasizing that mobilizing and organizing are both crucially important—and indeed interrelated—processes of effecting social change. Strategic organizing is always directed toward specific moments of mobilization that will force decision-makers’ hand, and moments of successful mobilization represent key opportunities for identifying and developing new organizers. A West Virginia teacher testifies: “[M]ore than anything, the strike changed people’s ideas of what is possible, I now have coworkers asking me about when we’re going to have a nationwide teachers’ strike, which I could have never imagined being uttered even a few months ago” (qtd. in Blanc, 2019, p. 97). This kind of experience is typical of involvement in large-scale social movements, and the effects can endure for a lifetime. A sizable empirical literature comparing people who did and did not participate in 1960s radical movements, for instance, demonstrates that participation had “dramatic and durable politicizing effect upon individuals” (Crossley, 2003, p. 50); as Crossley (2003, p. 50) writes: “participation in protest events or movements, whatever unrelated contingencies bring it about in the first instance, often creates a disposition towards further political activism; a ‘radical’ habitus.” I thus suggest that we think of mobilizing and organizing as the primary weapons of solidarity from below.^18^

To reiterate: the power elite have enormous wealth, political connections, media influence, and traditional weapons at their disposal. What we have is our numbers and necessity, which we can use to build the power of solidarity from below, and wield through the twin processes of mobilizing and organizing.

## Complications and clarifications

Before moving on, I want to elucidate my stance on certain features of this concept of solidarity, in anticipation of several obvious questions and concerns.^19^

First, and perhaps most pressingly, can solidarity from below be exhibited by conservative and reactionary groups that are opposed to progressive social change? To put it another way, is solidarity from below a *moralized* notion in the vein of Arendt, Scholz (2008), and others such that it is normatively legitimate by definition—or is it evaluatively neutral, as on the views of such theorists as Allen (1999) and Kolers (2016)?

In reply, let me make a few clarifications. Although I have (as Allen does) characterized solidarity from below as a specific type of normatively legitimate power-with, this legitimacy is not simply stipulated by definition as it is on her account. Instead, it arises from the fact that people acting in concert with the aim of improving their social conditions is always in principle a legitimate end in itself.^20^ So long as they are doing so freely (in the ordinary non-compatibilist sense) and sincerely in pursuit of a genuinely-held conception of justice, this collective activity as such is a worthy *basis* of power. However, the *goal* of some groups’ exercise of power may be deeply unjust; that is, the conception of justice held may be severely misguided. Thus, on my account, the January 6th protesters who stormed the White House in 2021 under the false impression that the election had been stolen from former President Trump (or that whites suffer from reverse racism, etc.) *might* perhaps^21^ count as exercising solidarity from below. But their doing so constitutes an abuse of that power. Similarly, and notoriously, corrupt labor officials or charismatic sexists may exploit their positions of influence, built atop the collective power of the masses, for self-serving ends that undermine the project of social change. In these cases, the normatively legitimate power of solidarity has been turned to wrongful ends.

So much can be said from within a pre-existing, independent normative framework that provides standards for distinguishing between morally good and evil ends. However, the connection between solidarity from below and moral goods is not wholly contingent. Because this particular form of power depends essentially on collaboration and collective action, it grows in proportion to the numbers of people involved in such a project. Hence, efforts to build solidarity from below that inherently contain exclusionary, hierarchical, or otherwise unjust features will eventually be limited, because of the way that solidarity from below is grounded in the power of numbers. No matter how many white Americans join such efforts, they will always be outnumbered—and hence potentially overpowered—by the greater solidarity from below that can be achieved^22^ by the global non-white majority.

A second question which follows naturally from the first is this: do we have *moral* or *prudential* reasons to engage others in building solidarity from below? Are these reasons categorical or hypothetical, that is, do they apply to everyone or only those antecedently committed to the project of social change?

In my view, both types of reasons are operative. In other work, I have argued that we each bear responsibility for addressing injustice in virtue of our social roles (Zheng, 2018), and I follow theorists such as Sally Haslanger (2017) and Clarissa Hayward (2017) in thinking that large-scale collective action—that is, the exercise of solidarity from below—is the most effective way to do so. This is a universal moral reason for joining with others to build solidarity from below. However, I think it is important to stress that there is also a prudential reason to do so, with a more limited scope: *if* one is antecedently committed to fighting some aspect of injustice, then one also has prudential reason to fight all its other aspects. This claim follows from what I have previously called the “intersectionality thesis,” the idea that we cannot successfully rectify any one oppression without overcoming all others (Zheng, 2018).

Clearly, the above claims require defense, which I cannot carry out here. There are further questions that present themselves, such as the following: How exactly is solidarity from below to be wielded so as to successfully bring about social change? What are the best ways to build and maintain solidarity from below, especially given the internal diversity within any social movement and the cross-cutting interests that often pit them against one another? When and how does such solidarity break down, and how can it be repaired? If solidarity is to be more than just a catchword but an actual way forward towards achieving transformative social change, then we will need answers to such questions. Here, I will content myself with a few concluding words elaborating on the relationship between solidarity from below and more traditional moral-relational concepts of solidarity.

## Conclusion

To think of solidarity as a form of collective power rather than a moral sentiment, relationship, or duty is in some sense to de-moralize the concept,^23^ to treat it as an instrumental good whose importance lies in its potential to effect social change. But this is not at all to deny that solidaristic relations and attitudes are also normatively valuable in themselves, a fact well-recognized by all the theorists who adopt this approach. It is also understandable that philosophers have mostly been interested in the distinctively normative and moral aspects of solidarity. In particular, they have analyzed how these normatively valuable relationships and attitudes give rise to distinctive motivations to act, or to moral obligations in which those in solidary relationships are obligated to lend material and moral support to those in need (like the good Samaritan) or struggle (whether this is between privileged ‘allies’ and those on the front lines, or between equal parties fighting on different fronts) (Shelby, 2005; Scholz, 2008; Kolers, 2016). Of course, the stronger the relational bonds and prosocial attitudes between individuals, the more gladly spontaneously will such solidary acts be undertaken. Shelby (2005), for instance, is especially concerned with identifying the right kind of attitude to serve as a motivation for solidary action: since he rejects racial identification as a basis for Black solidarity, he argues instead that a kind of empathy and enlightened self-interest empathy are sufficient for stimulating action. Thus, on many views, we should cultivate solidary relations and attitudes in order to reap the benefits of collective action that they will naturally produce.

To put a finer point on it: existing philosophical views take the moral-relational aspects of solidarity as logically and causally prior, understanding it as a distinctive kind of *motive* or *reason* for action—which can, secondarily, be activated by groups, movements, etc. interested in pursuing social change. By contrast, my approach reverses or at least challenges this order of emphasis. Logically, I begin with the feature of solidarity that makes it so vital for social movements seeking radical change: the fact that it is the only form of power available to the powerless. This is the primary reason why the oppressed and exploited are interested in solidarity. I thereby conceive of solidarity in the first place *as* a form of power: the moral-relational aspects of solidarity emerge secondarily from the process of collective power, and not the other way around.^24^ This de-moralization brings the concept of solidarity out of the ethical realm of duty, affect, and attitude into the realm of political philosophy, where issues of power are paramount. Building solidarity from below is not a purely altruistic action undertaken for other-regarding reasons; it is self-regarding, but where the ‘self’ is understood as deeply interrelated with or even partially constituted by the Other.

That being said, I readily acknowledge that the relationship between the moral-relational aspects of solidarity and its being a form of collective power is likely to be complex. What is clear is that the former can causally produce the latter, and vice versa, and that our theoretical interests may be rooted in either the ethical or the political. In keeping with solidarity’s use as bridge concept (see Sect. 1.2), it is perhaps more helpful to think of these as concepts of solidarity as being in inextricable and dialectical relationship, where neither side has priority.^25^

These are all empirical claims, to be sure. But let me advance here some evidence in favor of the perhaps more surprising direction, that is, the claim that building solidarity from below can generate moral sentiments, relationships, and duties where these were previously fraught or nonexistent. This is supported by a significant body of social psychological research demonstrating that intergroup conflict and prejudice is reduced when groups share “superordinate goals” to which they can all contribute, and for which they must interdependently rely on one other (Allport, 1954; Aronson et al., 1978; Deutsch, 1973; Johnson & Johnson, 1991; Sherif, 1958). Examples also abound in historical and contemporary struggles, from racial/ethnic animosity transmuted into interracial solidarity over the course of union campaigns spanning the 1930s to the 2010s (Brueggemann & Boswell, 1998; Horton et al., 1998; McAlevey, 2019b), or from the women, lesbian and gay activists’ support for striking miners in Thatcherite England to trans anarchists joining a 2019 coal miners blockade in eastern Kentucky (Ali, 1986; Kelliher, 2014; Lewis, 2019; Spence & Stephenson, 2009). As Horton (1998), who successfully organized an interracial mill workers’ union in South Carolina that won equal wages for Black and white workers after a 1937 strike, reflects:

Once people took the position of working on the basis of equality in their union, they had to face a lot of opposition from their rank and file members, their families and the Klan [as well as] society in general that opposed them…I think that we may all be mixed up psychologically, but I don’t think that we are going to solve our personal problems just by searching our souls or by getting a professional therapist to help us work out our internal, individual problems. I think these problems get resolved much faster in action, preferably in some kind of social movement…When people get involved in a movement, they must take sides, and in the struggle, individual problems become less important or disappear altogether. (92–93)

In other words, often the most effective way to cultivate intra- and intergroup feelings of solidarity is through the process of building the collective power of solidarity from below—and testing it in the actual experience of struggle (cf. Brueggemann & Boswell, 1998).

Of course, all of this is much easier said than done, and there are far more numerous examples of solidary efforts torn apart by racial, gender, class, and other divisions. Historically, many of the workers’ movements that popularized the notion of class solidarity did not include low-skilled, women, or non-white workers; conversely, many prominent feminist and antiracist movements have paid very little attention to class. McAlevey (2018) describes an interview she gave alongside an organizer of the first national Women’s March after the election of U.S. President Donald Trump, in which she pointed out that numerically the largest organizations of women in the country are trade unions, and that unions are the primary defenders of working-class women’s workplace rights. When asked whether the Women’s March would demand a mass expansion of trade unions, however, the organizer replied: “That’s actually not my topic.” McAlevey goes on to declare: “The idea that it’s ‘not my issue’ is what is wrong with the so-called progressive movement in this country.” Decades earlier, bell hooks (1984, p. 62) described the ‘silos’ in which disagreements over strategy and emphasis led feminists to splinter into socialist feminists concerned with class, Black women concerned with racism, and so on, writing: “Sexism, racism, and classism divide women from one another.” This is because they are what Cathy Cohen (1999) calls “cross-cutting political issues,” that is, “concerns which *disproportionately and directly* affect only certain segments of a marginal group.”

Indeed, solidarity from below—unlike that of weapons or wealth—dissipates as people cease to act collectively, because powerless individuals only potentially wield that power, which can only be achieved in collaboration with others. Hence, cross-cutting divisions are deliberately exacerbated by the powerful. Temptations to what W. E. B. Du Bois (1903/1990) called “the wages of whiteness” (or maleness, middle-classness, able-bodiedness, etc.) lead many individuals to identify aspirationally with groups above them, rather than recognizing the objective relations that align their actual interests with the more vulnerable and downtrodden beneath them.^26^ Yet various histories of collective struggle, I believe, give us reason to hope that these barriers to solidarity can be overcome. Though I do not have space to develop this idea, I follow Diarmaid Kelliher (2018) in thinking that work on intersectionality is critical here, especially when grounded in analyses of actual struggle (see, e.g., Tait, 2016; Clawson, 2003). As intersectional feminist theorists have long argued, the same differences that divide us can also be a source of strength, wisdom, and generative creativity (e.g., Lorde, 1981/2007; Reagon, 1981/2000; hooks, 1984; Yuval-Davis, 1994; Dean, 1996; Mohanty, 2003).

The power of solidarity, then, is not easy to unlock. But we have little hope of overcoming the devastating injustices that plague our world if we do not learn to do so. While ultimately this task can only be accomplished through concrete struggle on the ground, I hope that the conceptual work I have done here will aid in that task.

## Footnotes

1 To be sure, a nation-state is a circumscribed subset of humanity as a whole, and these attitudes and relationships are accordingly restricted to members of the same society or state. Nevertheless, within the borders of that general conception of “society,” the attitudes and relationships described here are meant to apply universally, regardless of group difference.

2 For discussion of the evolution of the concept in Habermas’ thought, see Carrabregu (2016) and Pierce (2018).

3 This list closely resembles Larry May’s (1996) five elements of solidarity: conscious group identification, bonds of sentiment, interest in the group’s well-being, shared values and beliefs, and readiness to show moral support (p. 44).

4 I am grateful to Kristie Dotson for discussion of this point.

5 For the social democratic strand of Marxism, see the discussion of Kautsky and Bernstein in section 1.2 above.

6 Marx and Engels also had a distinct conception of solidarity, conceived as the ideal social relations between people that were precluded by capitalism and could only obtain under communism (Stjernø, 2009). This is, in effect, their answer to the second question (section 1.2).

7 Scholz distinguishes political solidarity from “social solidarity” and “civic solidarity,” which are used to answer the other three questions I have outlined.

8 Kolers contrasts his account of solidarity with the views described here, which he calls “teleological” because they presuppose a shared goal, towards which solidarity is undertaken to achieve. By contrast, he argues, genuine solidarity—understood as a distinctive reason for action—essentially involves *deference* toward another group’s judgment and goals. Thus shared interests arise out of solidarity, not the other way around.

9 For instance, certain individual identities, e.g. “the homosexual,” only exist against a background of oppressive power, i.e. homophobia. In contexts such as ancient Greece, where same-sex relations are routinely accepted, individuals engaging in such behavior do not consider it a defining part of their identity, nor do others mark them out as forming a social category on that basis.

10 For more on the distinction between power-over and power-to in political theory, see Dowding (1991/2019) and Pansardi (2012); see also Holloway (2002). Development theorists such as Rowlands (1997) and Green (2016) add a fourth concept of *power-within*, which refers to an individual’s confidence in her own dignity, worth, rights, and entitlements.

11 Allen (1999) gives the example of a coach exercising power over her athletes for their benefit.

12 As critics of the notion of “empowerment” have noted, however, it is not uniformly positive (Bhattacharya, Arruzza, & Fraser, 2019; Yuval-Davis, 1994). Some agents may exercise power-to in ways that dominate others (Allen, 1999), e.g. when (white) middle-class women’s “empowerment” in the workplace depends on the exploitation of poor and working-class women of color. See section 2.2 for more on this case.

13 Others, however, follow Arendt in conceiving of power-with as legitimate by definition; indeed, they conceive of “empowerment” in terms of power-with rather than power-to (Pansardi, 2011).

14 I follow Young’s (1990, 37-38) helpful distinctions between injustice, domination, and oppression: for Young, “injustice” is to be understood *in terms of* the social-structural processes of “domination,” which prevents people from exercising control over and determining their own actions, and “oppression,” which prevents people from flourishing and developing themselves through exercise of expressive and social capacities. While Young takes “exploitation” to be one of five irreducible “faces” of oppression, I am more open to a (Marxian) tradition of thinking of exploitation as opposed to oppression, where they represent material vs. symbolic dimensions of domination. Let me note, also, that to say injustice is a systemic whole is *not* to deny that there is all variety of internal tensions and contradictions – indeed, these are precisely the ‘cracks’ that can be exploited to bring about change.

15 But see section 3 for some complications. Note also that when I say “normatively legitimate,” I mean that the power is *genuinely* legitimate and not merely (i.e., descriptively) believed to be legitimate.

16 Sharp (1973, p. 38) quotes a former U.S. Presidential aide as saying: “Half of a President’s suggestions, which theoretically carry the weight of orders, can be safely forgotten by a Cabinet member. And if the President asks about a suggestion a second time, he [sic] can be told that it is being investigated. If he asks a third time, a wise Cabinet officer will give him at least part of what he suggests. But only occasionally, except about the most important matters, do Presidents ever get around to asking three times.” I am grateful to Luke O'Sullivan for discussion of this point.

17 Note, however, that this metaphor is *not* a meant to be a social ontological model of how oppression and domination is actually structured. Moreover, it is important not to interpret ‘99% vs. 1%’ to mean a binary conflict between two actual demographic groups—rather, the phrase is shorthand for a *system* that needs to be abolished such that no individuals or groups stand in these relations to one another. I am indebted to Sally Haslanger for this latter point.

18 This does not mean, however, that they are the *only* weapons. Countercultural communities that develop alternatives to prevailing oppressive norms, for instance, may become highly influential in popular discourse, thereby challenging pernicious ideologies. I am indebted to Sally Scholz for this point.

19 I am indebted to Avery Kolers for extremely helpful discussion of many points in this section.

20 Solidarity from below is thus an expression of what Enrique Dussel (2008, pp. 14-15) describes as *potentia,* a “common-will-to-live” or a “convergence of wills toward a common good.” Such *potentia*, for Dussel, can never be taken away, for it is always and only held legitimately by the people. However, *potentia* must be manifest in the world—that is, it must be institutionalized into *potestas*—via an organized system comprising differentiated functions of governance, representation, decision-making, etc., and it is this schism between *potential* and *potestas* opens up the possibility for the abusive fetishization of power (i.e., the grasping of power for its own sake).

21 However, as in all such cases, there is reason to doubt the degree to which these efforts are truly exercises of solidarity from below, for popular movements that seek to retain the status quo are often funded and supported by powerful actors. See, e.g., Cho et al. (2011) on the detrimental effects of such “astroturfing” on genuine grassroots movements.

22 Achieving such power is by no means guaranteed, however, as I discuss briefly in section 4.

23 I am grateful to Sandra Field for this way of putting the point.

24 See previous footnote on Kolers (2016). My argument here invokes a parallel structure, albeit in a teleological rather than non-teleological direction.

25 I am indebted to Phoebe Mak Rui Teng, as well as an anonymous reviewer, for discussion of this point.

26 I am grateful to an anonymous reviewer for pushing me to elaborate these points, and for this way of conceptualizing the problem.
